# Attenuation of diabetic kidney injury in DPP4-deficient rats; role of GLP-1 on the suppression of AGE formation by inducing glyoxalase 1

**DOI:** 10.18632/aging.102643

**Published:** 2020-01-06

**Authors:** Mithun Kumer Sarker, Jong Han Lee, Dae Ho Lee, Kwang-Hoon Chun, Hee-Sook Jun

**Affiliations:** 1 College of Pharmacy and Gachon Institute of Pharmaceutical Science, Gachon University, Incheon, Republic of Korea; 2 Lee Gil Ya Cancer and Diabetes Institute, Gachon University, Incheon, Republic of Korea; 3 Department of Internal Medicine, Gil Medical Center, Gachon University College of Medicine, Incheon, Republic of Korea; 4 Gachon Medical and Convergence Institute, Gil Medical Center, Incheon, Republic of Korea

**Keywords:** diabetic nephropathy, dipeptidyl peptidase 4, glucagon-like peptide-1, glyoxalase-1, advanced glycation end products

## Abstract

Dipeptidyl peptidase 4 (DPP4) inactivates incretin hormone glucagon-like peptide-1. DPP4 inhibitors may exert beneficial effects on diabetic nephropathy (DN) independently of glycemic control; however, the mechanisms underlying are not fully understood. Here, we investigated the mechanisms of the beneficial effects of DPP4 inhibition on DN using DPP4-deficient (DPP4-def) rats and rat mesangial cells.

Blood glucose and HbA1c significantly increased by streptozotocin (STZ) and no differences were between WT-STZ and DPP4-def-STZ. The albumin level in urine decreased significantly and the albumin/creatinine ratio decreased slightly in DPP4-def-STZ. The glomerular volume in DPP4-def-STZ significantly decreased compared with that of WT-STZ. Advanced glycation end products formation, receptor for AGE (RAGE) protein expression, and its downstream inflammatory cytokines and fibrotic factors in kidney tissue, were significantly suppressed in the DPP4-def-STZ compared to the WT-STZ with increasing glyoxalase-1 (GLO-1) expression responsible for the detoxification of methylglyoxal (MGO). *In vitro*, exendin-4 suppressed MGO-induced AGEs production by enhancing the expression of GLO-1 and nuclear factor-erythroid 2 p45 subunit-related factor 2, resulting in decreasing pro-inflammatory cytokine levels. This effect was abolished by GLO-1 siRNA.

Our data suggest that endogenously increased GLP-1 in DPP4-deficient rats contributes to the attenuation of DN partially by regulating AGEs formation via upregulation of GLO-1 expression.

## INTRODUCTION

The prevalence of diabetes is increasing worldwide, resulting in a dramatic increase in diabetic complications. Diabetic nephropathy (DN) is a complication of diabetes, and around 40% patients with diabetes ultimately develop DN [1]. Methylglyoxal (MGO), a reactive glucose metabolite, is produced in the glycolytic pathway, which is positively correlated with blood glucose levels [2, 3]. MGO is considered a main endogenous precursor for advanced glycation end products (AGEs) [4]. In fact, MGO is elevated in patients with diabetes and those with renal failure [5, 6]. Numerous studies have demonstrated that interactions between AGEs and their receptor (RAGE) evoke oxidative stress and the expression of inflammatory cytokines and fibrotic factors, leading to alterations in the renal structure and loss of renal function in diabetes [7–9]. RAGE knockout mice were resistant to the development of DN induced by streptozotocin (STZ) [10], suggesting that suppression of the AGE-RAGE axis in the kidneys might be a potential therapeutic target for treatment of DN.

Glucagon like peptide-1 (GLP-1) is a 30 amino acid long peptide hormone released from the lumen of digestive tract. It is known as an incretin hormone because of its role in enhancing the secretion of insulin [11–13]. Therefore, increasing the endogenous level of GLP-1 could be a plausible therapeutic approach for improving the glycemic control of type 2 diabetes. GLP-1 is rapidly metabolized by dipeptidyl peptidase-4 (DPP4) and has an exceedingly short half-life. Numerous studies on both human and animal models have shown that the activity of circulating DPP4 is increased in obesity and diabetic condition. Its inhibition has potentially beneficial effects in diabetes and diabetic disorders, including diabetic nephropathy [14–16]. Beyond its effect on glycemic control, accumulating evidence indicates a broader range of physiological roles including those in the regulation of autophagy, elevation of anti-inflammatory effects, as well as in the promotion of metabolic reprogramming of carbohydrate or lipid metabolism [17, 18].

DPP4 is known as adenosine deaminase complexing protein 2 or CD26 and is responsible for degrading incretin hormones, such as GLP-1 [19]. DPP4 also plays a pathogenic role in fibrosis development in various organs, particularly the kidney and liver [20–22]. DPP4 inhibition improves metabolic control by GLP-1-mediated insulin secretion in the pancreas and suppresses gluconeogenesis in the liver [22]. Accumulating evidence suggests that DPP4 inhibitors may prevent the onset and progression of DN beyond the effect by glycemic control [23, 24]. In addition, Matsui et al. recently showed that DPP4 deficiency attenuates DN partly by suppressing AGE-RAGE-induced oxidative stress [25]. However, the molecular mechanism by which DPP4 inhibition regulates the AGE-RAGE axis in DN remains poorly understood. In the current study, we investigated whether increased GLP-1 in DPP4-deficient rats attenuates DN by regulating AGEs formation and the mechanisms underlying this attenuation both *in vitro* and *in vivo.*

## RESULTS

### DPP4 deficiency attenuates albuminuria and recovers the altered glomerular structure in STZ-induced diabetic rats

**Figure 1:**
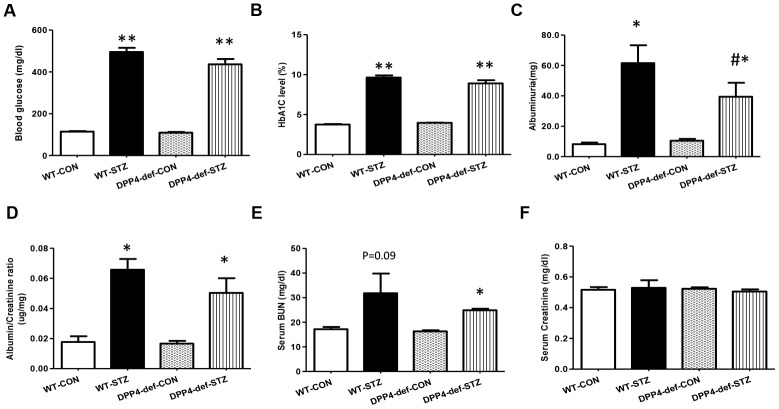
**DPP4 deficiency attenuates albuminuria in STZ-induced diabetic rats.** Both wild-type and DPP4-deficient rats were administered with IP injection at 30 mg/kg/day STZ three times. All samples were collected and evaluated as described in Materials and Methods. (**A**) Blood glucose level after 4 h fasting, (**B**) HbA1c level, (**C**) Albuminuria level, (**D**) Albumin/creatinine ratio. (**E**) Serum BUN level, (**F**) Serum creatinine level. WT-CON: wild-type control, WT-STZ: wild-type-STZ, DPP4-def-CON: DPP4-deficient control, DPP4-def-STZ: DPP4-deficient-STZ. Data are shown as the means ± SEM. *p < 0.05, ** p < 0.01 and WT-CON, *^#^p* < 0.05 and WT-STZ, n = 7–8 per group.

The blood glucose level was measured every week after STZ administration and the rats with blood glucose levels of more than 300 mg/dL were used for the experiments. Blood glucose and HbA1c levels were significantly increased in WT-STZ diabetic rats and there were no differences between WT and DPP4 deficient rats (Figure 1A, 1B). To investigate whether DPP4 deficiency affects the development of DN in STZ diabetic rats, we first examined urine albumin, BUN, and creatinine levels. Albumin in urine was significantly decreased in DPP4-def-STZ rats compared with that in WT-STZ rats. The albumin/creatinine ratio (ACR) was increased by STZ, and ACR showed reduction trend in DPP4-deficient diabetic rats compared to that in WT-STZ rats (Figure 1C, 1D). Since BUN and creatinine are biomarkers for renal dysfunction, we also measured their levels in the serum. Similarly, BUN levels slightly increased in WT-STZ diabetic rats but did differ significantly between WT-STZ and DPP4-def-STZ rats (Figure 1E). Creatinine levels were similar among all groups (Figure 1F).

Water intake and urine volume were significantly elevated in both wild type (WT) and DPP4 deficient diabetic rats. The water intake was significantly decreased in the DPP4-def-STZ rats, and urine volume were also reduced, although not significant (Table 1).

**Table 1:** Effect of DPP4 deficiency on physiological parameters of STZ-induced diabetic rats.

**Characteristic**	**WT-CON**	**WT-STZ**	**DPP4-def-CON**	**DPP4-def -STZ**
Food intake (g/g per day)	0.032±0.01	0.100 ± 0.01^**^	0.054 ± 0.01^**, ##^	0.093 ± 0.01^**^
Water intake (ml/g per day)	0.097 ±0.01	0.413 ± 0.01^**^	0.104± 0.01 ^##^	0.327 ± 0.01^**, ##^
Urine volume (ml/g per day)	0.028±0.003	0.286 ± 0.017^**^	0.029 ± 0.002 ^##^	0.243 ± 0.019^*^
Body weight (g)	305.75± 4	223.71 ± 6^**^	279.86 ± 6^**,^^##^	207.43 ± 8^**^
Triglyceride (mg/dL)	39.49±1.693	107.51 ±19.933^**^	26.30± 1.589**^, ##^	32.61 ±4.571^, ##^
Cholesterol (mg/dL)	248.43±7.525	253.05 ±10.934	205.29 ±2.634**^,^^#^	239.72±4.527
Kidney weight (g)	6.90± 0.08	11.01 ± 0.24^*^	6.86 ± 0.21	10.41 ± 0.57*

**Figure 2:**
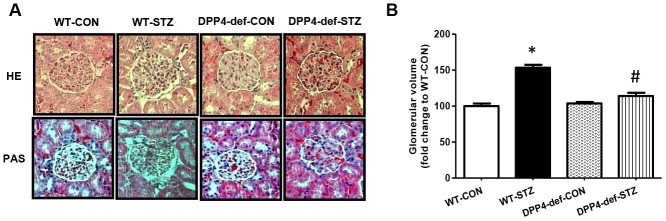
**DPP4 deficiency recovers the structure of glomerulus impaired by STZ.** Kidney samples were collected at 42 days, since over 300 mg/dL of blood glucose after STZ injection as described in the Materials and Methods. The glomerular volume was measured using the ImageJ software for at least 15 images from each kidney section. (**A**) Representative image of glomerulus by H&E staining and by PAS staining, (**B**) Glomerular volume. WT-CON: wild-type control, WT-STZ: wild-type-STZ, DPP4-def-CON: DPP4-deficient control, DPP4-def-STZ: DPP4-deficient-STZ. Data are shown as the means ± SEM. *p< 0.05 and WT-CON, *^#^p* < 0.05 and WT-STZ, n = 7–8 per group.

Glomerular matrix expansion is a hallmark of DN in the kidney [26, 27]. Therefore, we also examined whether DPP4 deficiency affects the expansion of the glomerular area in STZ-induced diabetic rats using hematoxylin and eosin or Periodic Acid–Schiff staining (Figure 2A). The kidney weight significantly increased in diabetic rats but was not significantly different between WT-STZ and DPP4-def-STZ (Table 1). The glomerular volume and glomerular tuft area were significantly increased in diabetic WT-rats, whereas these increases were remarkably reduced in DPP4 def-STZ rats (Figure 2B).

### Expression of TGF-β, fibronectin, and inflammatory cytokine is decreased in the kidney of DPP4-deficient diabetic rats

**Figure 3:**
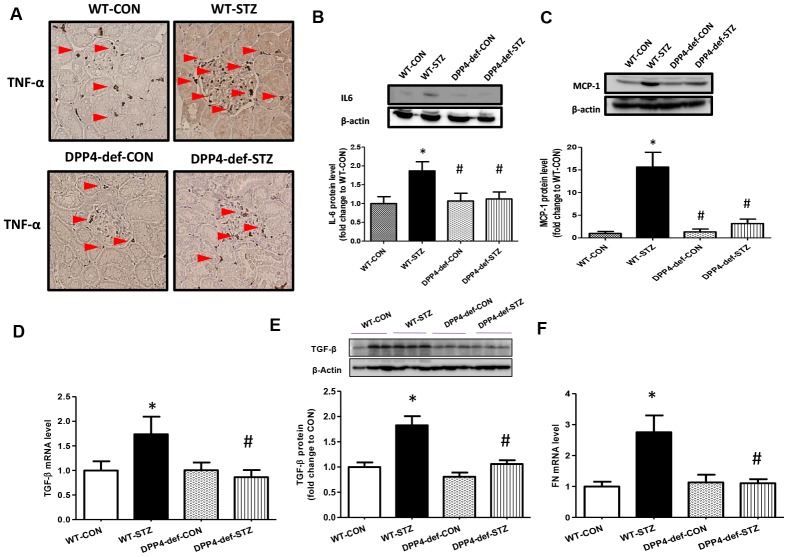
**The expression of inflammatory cytokines and fibrotic factors are reduced in STZ-induced diabetic rats.** (**A**) The kidney tissues were fixed in formalin and then subjected to immunofluorescence detection of TNF-α (arrow heads pointing to dark-brown dots indicating TNF-α expression). n = 5 per group, (**B**) IL6 protein level (**C**) MCP1 protein level (**D**) *TGF-β* mRNA level, (**E**) TGF-β protein level with a representative blot, (**F**) *Fibronectin* (FN) mRNA level in kidney tissues. Data are shown as the means ± SEM *p < 0.05 vs. WT-CON; *^#^p* < 0.05 vs. WT-STZ. n = 5–8 per group.

To examine whether there is a change in the expression of inflammatory factors and fibrotic factors in the kidney of DPP4-deficient diabetic rats, we evaluated the expression of tumor necrosis factor (TNF)-α, interleukin (IL6), and monocyte chemoattractant protein (MCP)-1 as inflammatory cytokines, and TGF-β and fibronectin (FN) as fibrotic factors. We found that the levels of TNF-α, IL6 and MCP-1 were significantly increased in WT diabetic rats. However, this increase was significantly inhibited in DPP4-def-STZ rats (Figure 3A–3C, Supplementary Figure 1). Consistently, TGF-β and FN expression was also increased in WT-STZ rats compared to that in WT-CON rats and was significantly inhibited in DPP4-def-STZ rats (Figure 3D–3F). Interestingly, the expression levels of TNF-α, IL6, MCP-1, and TGF-β were comparable with the respective levels in DPP4-def-CON rats (Figure 3, Supplementary Figure 1).

### AGE and RAGE expression is decreased and GLO-1 expression is increased in the kidney of DPP4-deficient diabetic rats

**Figure 4:**
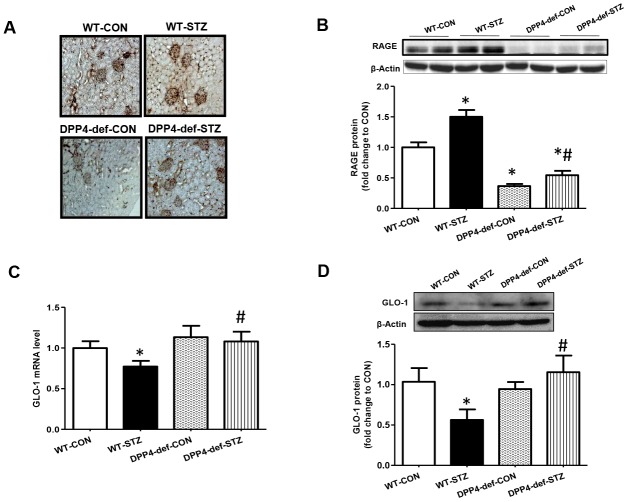
**Increased of AGE formation, RAGE and GLO-1 expression are inhibited in the kidney of DPP4 deficient diabetic rats**. Kidney samples were collected at 42 days, since over 300 mg/dL of blood glucose after STZ injection as described in Materials and Methods section. AGEs formation was evaluated using antibody against AGEs in the kidney section. Brown color indicates AGEs formation in staining. (**A**) AGEs formation, (**B**) RAGE protein level with representative blot (**C**) *GLO1* mRNA level (**D**) GLO-1 protein level with a representative blot in tissues. WT-CON: wild-type control, WT-STZ: wild-type-STZ, DPP4-def-CON: DPP4-deficient control, DPP4-def-STZ: DPP4-deficient-STZ. Data are shown as the means ± SEM. *p < 0.05 and WT-CON, *^#^p* < 0.05 and WT-STZ, n = 7–8 per group.

AGEs is a risk factor for the development of DN through their receptor, RAGE [28]. The interaction of AGE and RAGE induces the expression of pro-inflammatory cytokines and fibrotic factors [29, 30]. Thus, we first evaluated whether AGE formation is increased in diabetic rats. AGE formation dramatically increased in WT-STZ rats and this increase was ameliorated in DPP4 def-STZ rats (Figure 4A). In line with AGEs formation, the expression of RAGE was significantly increased in WT-STZ rats, but this increase was blocked in DPP4-def-STZ rats (Figure 4B). DPP4 deficiency itself also decreased RAGE expression compared to WT-CON (Figure 4B). GLO-1 catalyzes MGO produced from high glucose into *S*-lactoylglutathione, thereby reducing AGEs formation of DN risk factor [29]. Therefore, we examined the mRNA and protein expression levels of GLO-1 in the kidney tissues of diabetic rats. GLO-1 mRNA and protein expression were significantly decreased in the WT-STZ rats compared to in those in control rats, whereas the expression of GLO-1 was significantly increased in DPP4-def-STZ rats (Figure 4C, 4D).

### Ex-4 treatment reduces MGO-induced AGE formation and RAGE expression and increases GLO-1 expression in rat mesangial cells

**Figure 5:**
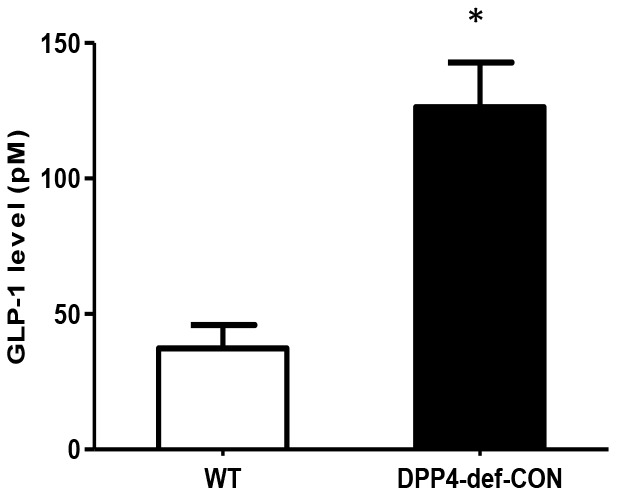
**Circulating plasma GLP-1 level is increased in DPP4-deficient rats.** Plasma GLP-1 concentration was measured using rat-specific GLP-1 ELISA kit within 3 h after collecting blood from wild-type and DPP4-deficient rats at 8 weeks of age. Data are shown as the means ± SEM. *p < 0.05 and WT, n = 4–6 per group.

**Figure 6:**
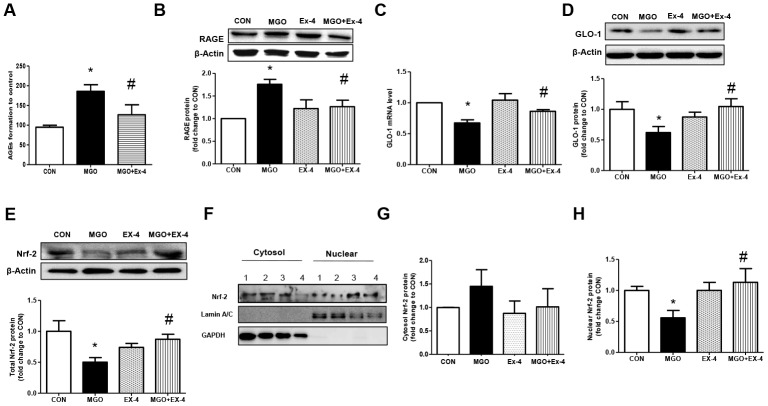
**Ex-4 treatment reduces MGO-induced AGEs formation and RAGE expression by upregulating GLO-1 enzyme and recovers the decrease in MGO–induced GLO-1 expression in rat mesangial cells.** Rat mesangial cells were treated either with 1 mM MGO, 10 nM Ex-4, or both for 10 h after synchronization with 1% fetal bovine serum for 13-16 h. AGEs formation was measured as described in Materials and Methods. (**A**) AGEs formation, (**B**) RAGE protein level with representative blot, (**C**) *GLO-1* mRNA level, and (**D**) GLO-1 protein level with a representative blot (**E**) Nrf-2 protein level with a representative blot in total protein extracts, (**F**) Representative blot of Nrf-2 protein in cytosol and nuclear fractions in rat mesangial cells. 1: CON; 2: MGO; 3: Ex-4; 4: MGO + Ex-4 (**G**) Nrf-2 protein level in cytosol fraction. (**H**) Nrf-2 protein level in nuclear fraction. Data are shown as the means ± SEM. *p < 0.05 and CON, *^#^p* < 0.05 and MGO, n = 4–7.

We first checked whether DPP4 affects GLO-1 expression. Recombinant DPP4 treatment of rat mesangial cells did not induce GLO-1 protein expression (Supplementary Figure 2). Serum GLP-1 levels were significantly increased in DPP4-deficient rats compared to those in WT rats (Figure 5). Therefore, we investigated whether GLP-1 contributes to the decrease in AGEs formation. We examined the effect of Ex-4, a GLP-1 receptor agonist, on MGO-induced AGEs formation in rat mesangial cells. MGO treatment increased AGEs formation by approximately 1.8-fold compared to that in the control without MGO treatment. However, AGEs formation was suppressed in the presence of Ex-4 (Figure 6A), and the final products of MGO detoxification system, D-lactate, significantly increased (Supplementary Figure 3). In addition, RAGE Metabolic cage study was conducted at 35-37 days since over 300 mg/dL of blood glucose after last STZ injection; other parameters collected at 42 days since over 300 mg/dL of blood glucose after last STZ injection. Data are shown means ± SEM; WT-CON: wild type control, WT-STZ: wild type-STZ, DPP4-def-CON: DPP4 deficient control, DPP4-def-STZ: DPP-4 deficient-STZ. *p < 0.05, **p < 0.01 and WT-CON, ^#^P < 0.05, ^##^P < 0.01 and WT-STZ n= 7-8 per group.

protein expression was significantly increased by MGO treatment but reduced to basal levels following Ex-4 treatment. However, Ex-4 itself did not affect RAGE expression (Figure 6B). In rat mesangial cells exposed to MGO, the GLO-1 mRNA and protein expression levels were reduced by around 30% and 40%, respectively; however, these reductions were significantly reversed by Ex-4 treatment as shown in Figure 6C, 6D. Since Nrf-2 directly regulates the transcription of GLO-1 [31], we also investigated whether Ex-4 affects Nrf-2 expression and its activation. Ex-4 treatment led to increased Nrf-2 protein expression and induced its translocation from the cytosol into the nucleus in the presence of MGO (Figure 6E–6H), indicating that Ex-4 treatment induces Nrf-2 activation.

### Ex-4 treatment reduces the MGO-induced expression of inflammatory cytokines in rat mesangial cells

**Figure 7:**
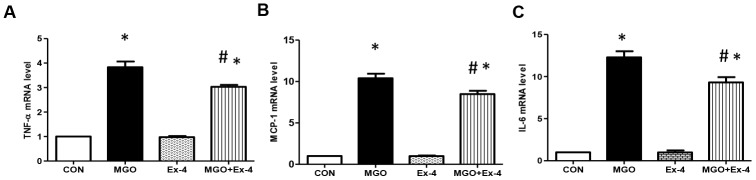
**Ex-4 treatment reduces MGO-induced inflammatory cytokine expression in rat mesangial cells.** Rat mesangial cells were treated either with 1 mM MGO, 10 nM Ex-4, or both for 10 h after synchronization with 1% fetal bovine serum for 13-16 h. (**A**) *TNF-α* mRNA level, (**B**) *MCP-1* mRNA level, (**C**) *IL6* mRNA level in rat mesangial cells. Data are shown as the means ± SEM. *p < 0.05 and CON, *^#^p* < 0.05 and MGO, n = 3–4.

AGEs bind RAGE and induce reactive oxygen species production and inflammatory cytokine expression [8]. As Ex-4 treatment reduced MGO-induced AGEs formation, we evaluated whether Ex-4 reduces MGO- induced expression of inflammatory cytokines. In line with RAGE expression, Ex-4 itself did not affect inflammatory cytokine expression (Figure 7A–7C). However, when we treated rat mesangial cells with MGO, the expression of TNF-α, MCP-1, and IL-6 mRNA was remarkably elevated but decreased by Ex-4 treatment (Figure 7A–7C).

### Effect of Ex-4 on the reduction of AGEs formation and inflammatory cytokine expression is abolished in the knockdown of GLO-1 in rat mesangial cells

**Figure 8:**
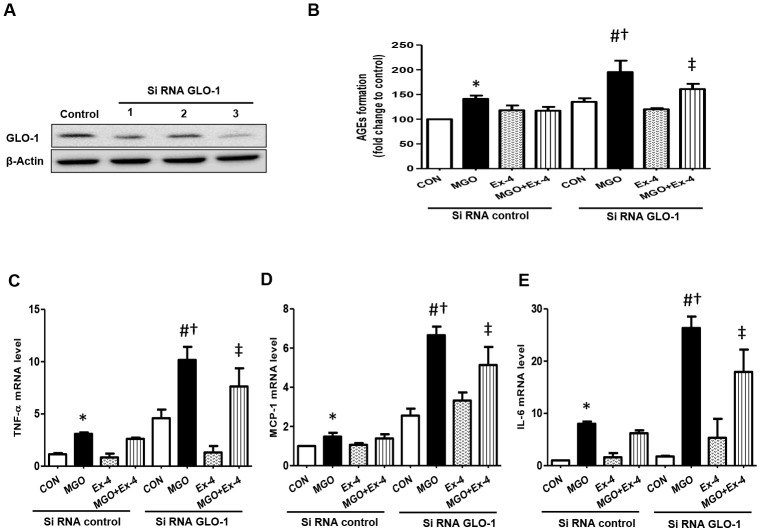
**AGEs formation and inflammatory cytokines are further increased in the knockdown of GLO-1**. Rat mesangial cells were transfected either with siRNA control or siRNA GLO-1 and, then, treated with 0.75 mM MGO and 10 nM Ex-4 for 4 h. (**A**) siRNA GLO-1 transfection efficacy. (**B**) AGEs formation level in the knockdown of GLO-1 and mRNA expression levels of inflammatory cytokines including (**C**) *TNF-α* (**D**) *MCP-1*, and (**E**) *IL-6*. Data are shown as the means ± SEM. *p < 0.05 *vs.* siRNA CON, ^#^*p* < 0.05 *vs*. siRNA CON + MGO, ^†^*p* < 0.05 *vs*. siRNA GLO-1 + CON, ^‡^*p* < 0.05 *vs.* siRNA CON + MGO + Ex-4. n = 5–6.

To investigate whether Ex-4 directly inhibits AGEs formation by regulating GLO-1, we knocked down GLO-1 in rat mesangial cells using siRNA GLO-1 as confirming knockdown at the protein level (Figure 8A). AGEs formation was significantly induced by MGO treatment in scrambled siRNA transfected cells and further increased in GLO-1 siRNA transfected cells (Figure 8B). Ex-4 treatment suppressed MGO-induced AGEs formation, but was less effective in the knockdown of GLO-1 and the increased level was comparable to that by MGO treatment in the scrambled control (Figure 8B). In agreement with these data, the expression of inflammatory cytokines, such as TNF-α, IL-6, and MCP-1, showed similar trends as AGEs formation between the scrambled control and GLO-1 siRNA-transfected cells treated with MGO (Figure 8C–8E). All cytokines were highly increased under GLO-1 knockdown conditions compared to under scrambled conditions, and the suppressive effect of Ex-4 against cytokine expression was inhibited in the knockdown of GLO-1 (Figure 8C–8E).

## DISCUSSION

Numerous studies on both human and animal models showed that circulating DPP4 activity is increased [32, 33] and its inhibition has a potential beneficial effects in diabetes and diabetic disorders [19, 22]. DPP4 inhibitors (such as linagliptin and DA-1229) suppressed TGF-β/Smad-mediated renal fibrosis [34, 35] and prevented podocyte damage without lowering the blood glucose in diabetic condition [24, 36], [37]. DPP4 deficiency also protects kidney from acute ischemia reperfusion injury, indicating that DPP4 deficiency contributes to the attenuation of DN [38]. Recently, Matsui T. et al reported that the levels of renal AGE-modified protein, oxidative stress and ICAM-1 gene mRNA expression were decreased and reduced proteinuria in DPP4 deficient diabetic rats [25]. However, the underlying mechanism by which AGE-modified proteins are suppressed has not been investigated. In the current study, we showed that the endogenously increased GLP-1 *in vivo* due to DPP4 deficiency upregulated GLO-1 expression and increased MGO detoxification, resulting in reduced AGEs formation.

Histological analysis showed that the glomerular volume and mesangial matrix expansion were increased in diabetic rats and significantly reduced in DPP4-deficient diabetic rats. However, our biochemical analysis of the serum and urine did not significantly reflect these histological improvements such as creatinine and ACR, but showed a reduction in the albumin level in urine and decreased triglyceride in serum. Similarly, Moellmann et al. reported that overexpression of a GLP-1 mutant, resistant to DPP4, showed renoprotective effects, such as reduced glycosuria and inflammation without reducing proteinuria in the kidney of STZ-induced diabetic mice [39]. In contrast, Matsui et al. showed deceased fibrosis and improved kidney functions in DPP4-deficient rats, including reduced ACR [25]. These discrepancies among different studies, including ours, may be due to the variance in severity of renal pathology of recruited animals. Hyperglycemia elevates MGO production from glycolysis, which is a by-product of glycolysis and main source of AGEs formation [40]. AGEs accumulation induces expression of its receptor (RAGE). Indeed, RAGE expression is strongly correlated with the plasma levels of AGEs [41]. Activation of AGE-RAGE signaling is considered one of the main mechanisms involved in the development of DN [28, 42]. Hou et al. showed that elevated RAGE amplifies AGEs-induced monocyte perturbation and contributes to the monocyte-mediated systemic inflammatory response in chronic kidney disease [41]. Increased production of AGEs and its interaction with its receptor (RAGE) also evoke oxidative stress generation, inflammatory cytokines and fibrotic factors expression, thereby leading to alterations in the renal structure and loss of renal function in diabetic conditions [9, 10].

To reach similar hyperglycemic states between WT and DPP4-deficient rats, we maintained the rats until having similar blood glucose and HbA1c levels, indicating physiologically similar induction levels of AGEs formation. Our data showed that AGEs formation in the kidney was significantly reduced in the DPP4-deficient diabetic rats compared to that in WT diabetic rats. Since DPP4 protein levels were not significantly different between WT-CON and DPP4-def-CON rats (data not shown) and serum GLP-1 levels were significantly increased in DPP4-deficient rats, the reduction in AGE formation may be attributed to the increased GLP-1 levels. Although DPP4 deficiency itself improves the physiological parameters (body weight and triglyceride) monitored in the current study, we did not observe any alteration in the renal structure as well as differences in the inflammatory cytokine and GLO-1 expression levels between WT and DPP4 deficient rats under normal physiological conditions. Moreover, recombinant DPP4 treatment itself did not induce GLO-1 protein expression either with or without MGO. These data suggested that increased GLP-1 expression might be a main contributor in the regulation of GLO-1 expression and AGE formation. However, Kaifu K et al. recently reported that AGE treatment failed to stimulate the NF-kB signaling pathway in tubular cells isolated from DPP4 deficient rats unlike in tubular cells isolated from control rats, suggesting an autocrine effect of DPP4, such as the decrease in RAGE protein expression under DPP4 deficiency, as shown in our current study [43].

In response to AGEs formation, the RAGE protein level was increased significantly in the WT diabetic rats. Interestingly, RAGE protein was barely detectable in DPP4-deficient rats but was significantly induced by STZ treatment. These data revealed a clear correlation between AGEs production and RAGE expression in *in vivo*. Additionally, our *in vitro* study using rat mesangial cells, exposed to MGO, recapitulated this observation in diabetic rats. Ex-4 treatment suppressed RAGE protein expression induced by MGO, as observed in DPP4-deficient diabetic rats. Similar to our data, those reported in other studies showed that Ex-4 attenuates rat mesangial cell dysfunction caused by high-glucose exposure through the AMPK pathway and suppresses renal AGE-modified protein formation in STZ-induced diabetic rats [25, 44]. Moreover, RAGE knockout mice were resistant to the development of DN induced by STZ [10]. Taken together, these data suggested that AGE and RAGE signaling activation is regulated by GLP-1/Ex-4.

AGE/RAGE-mediated inflammatory cytokines and fibrotic factors expression play an important role in the development of DN [45]. Our results showed that the mRNA expression levels of TNF-α and MCP-1 were significantly increased in the WT-STZ rats compared to in the WT-CON rats but their expression levels were significantly suppressed in the DPP4-def-STZ rats. Consistently, DPP4 deficiency decreased the expression levels of fibrotic factors (TGF-β and fibronectin) elevated by STZ administration. In addition, TNF-α, IL-6, and MCP-1 expression was significantly inhibited by Ex-4 treatment in rat mesangial cells exposed to MGO, suggesting that GLP-1/Ex-4 might inhibit inflammatory cytokine and fibrotic factor expression by suppressing AGEs formation.

The formation of AGEs and the upregulation of their downstream cytokines have been suggested as potential mechanisms involved in the development of DN [46, 47]. Thus, reducing MGO levels may be beneficial in preventing the pathogenesis of DN. Endogenous MGO levels can be reduced by a detoxification process involving the glutathione-dependent GLO pathway. MGO spontaneously reacts with glutathione, thereby forming a D-lactoylglutathione, which is subsequently metabolized to D-lactate by GLO-1 and GLO-2 [48]. Our current results showed that GLO-1 protein and mRNA expression in the kidney tissues was significantly decreased in the WT-STZ rats compared to in the WT-CON rats, but its expression significantly recovered to the level of control group in DPP4-deficient rats. Ex-4 treatment inhibited MGO-induced AGEs formation and inflammatory cytokine expression, whereas these inhibitory effects of Ex-4 were abolished in GLO-1 knockdown cells. These results suggested that GLP-1/Ex-4 increases MGO detoxification by upregulating GLO-1 expression. Consistent with our data, overexpression of GLO-1 in apolipoprotein E-null mice prevented albuminuria in the STZ-induced diabetic condition [49], whereas knockdown of GLO-1 induced DN even in non-diabetic mice [50]. Moreover, GLO-1 overexpression completely prevented DN without altering the hyperglycemic condition [49].

Recently, it was reported that MGO-induced AGEs formation is reduced by nuclear factor-erythroid 2 p45 subunit-related factor-2 (Nrf-2)-mediated upregulation of GLO-1 expression [51, 52]. Ex-4 has been reported to activate Nrf-2 in a pancreatic beta-cell line [53]. Similarly, our data also showed that Ex-4 treatment increases Nrf-2 protein expression and induces its translocation from cytosol to nucleus in the presence of MGO. Taken together, these data suggested that GLO-1 expression may be upregulated via activation of Nrf-2 by GLP-1/Ex-4.

**Figure 9:**
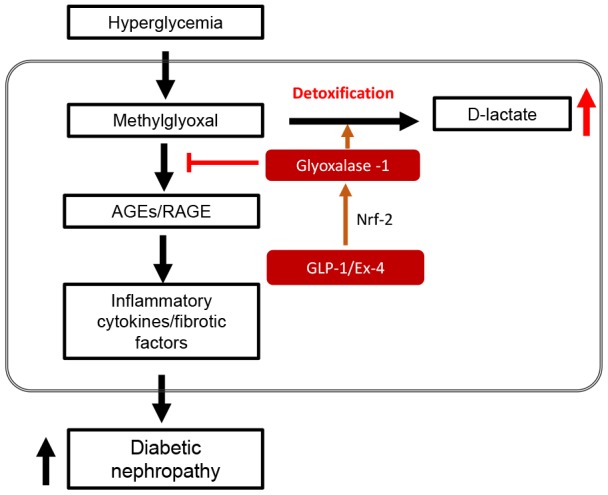
**Schematic diagram in STZ-induced diabetic nephropathy showing GLP-1/Ex-4 increases detoxification of methylglyoxal (MGO) through the regulation of glyoxalase-1.** Hyperglycemia-induced MGO accumulation under diabetic condition activates the AGEs-RAGE signaling pathway, which results in diabetic nephropathy through upregulation of the expression of inflammatory cytokines and fibrotic factors. In contrast, GLP-1/Ex-4 enhances detoxification of MGO, producing D-lactate through the regulation of glyoxalase-1 expression. GLP-1: Glucagon like peptide-1, Ex-4: Exendin-4, Nrf-2: Nuclear factor-erythroid 2 p45 subunit-related factor-2, AGEs: Advanced glycation end products, RAGE: Receptor AGE.

In conclusion, our results showed that STZ-induced diabetic hyperglycemia impaired kidney structure and function through AGEs-RAGE mediated pro-inflammatory cytokines and fibrotic factor expression. Endogenously increased GLP-1 expression in DPP4-deficient rats decreased AGEs formation by upregulating GLO-1, contributing to the recovery of the impaired kidney structure and function by downregulating pro-inflammatory cytokine and fibrotic factor expression as shown in Figure 9.

## MATERIALS AND METHODS

### Animals

DPP4 deficient (def) rats were purchased from Rat Resource and Research Center at the University of Missouri. Six-week-old male Fischer 344 wild-type (WT) rats were purchased from Charles River Laboratories (Wilmington, MA, USA). The rats were randomly divided into four experimental groups as follows: wild-type control (WT-CON), wild-type STZ (WT-STZ), DPP4 deficiency-control (DPP4-def-CON), and DPP4 deficiency-STZ (DPP4-def-STZ). To induce diabetes, 8-week-old rats in all STZ groups were administered 30 mg/kg /day STZ (Streptozotocin, Sigma, S0130) intraperitoneally (i.p) after 4 hours (h) fasting in the morning 3 times. As a control, the same volume of citrate buffer (pH 4.5) was injected. All animal care and treatments were conducted in accordance with the guideline for the animal use and care committee of the Gachon University and Lee Gil Ya diabetes and cancer institute. Blood glucose from the tail vein blood was measured every week until the end of the experiment using a glucose analyzer (One Touch^®^Ultra, Lifescan Johnson and Johnson, Milpitas, CA) after 4 h of fasting in the morning. The level of hemoglobin A1c (HbA1c) in blood was assessed before STZ injection and at the end of experiments using a DCA System HbA1c Reagent Kit (SIEMENS, New York, USA). At 42 days since over 300 mg/dL of blood glucose after the last STZ injection, we collected blood and kidney (L/R) samples right after measuring body weight for further analysis. The tissue samples were stored at -80°C until use. The levels of cholesterol and triglyceride in serum were determined by commercially available kits (#AM203, ASAN HDL-Cholesterol; #AM157K, ASAN TG-s, ASAN) according to manufacturer’s instructions.

### Measurement of biochemical parameters in blood and urine

The rats were placed in individual mouse metabolic cages for 24 h at 35–37 days since over 300 mg/dL of blood glucose after the last STZ injection. Food intake, water intake, and urine volumes were measured. The blood urea nitrogen (BUN), creatinine and micro-albumin were measured in serum collected at end of experiment or urine from metabolic cage using a biochemical analyzer (Beckman, USA).

### Renal histological assessment

The rats were sacrificed and rapidly fixed in 10% formalin buffer and embedded in paraffin. Next, 3-μm sections were stained with hematoxylin and eosin to observe alterations in the kidney structure or Periodic acid-Schiff to assess basement membrane thickening and glomerular volume. Glomerulus images were acquired by light microscopy at 200x magnification. Glomerular volumes were analyzed with the ImageJ program (NIH, Bethesda, MD, USA) for up to 15 glomeruli in each rat. To assess AGEs or the protein expression level of anti-tumor necrosis factor (TNF)-α, we also stained the sections with a specific antibody for AGEs (#ab23722, Abcam, Cambridge, UK) or TNF-α (sc-1350, Santa Cruz) and then visualized the sections using the DAB substrate chromogen system (K346811, Dako, Glostrup, Denmark).

### Measurement of plasma GLP-1

Blood samples were collected from 8-week-old WT and DPP4-deficient rats. Plasma GLP-1 levels were measured within 3 h of collecting the blood with a Rat ELISA kit (MBS2501740, MyBioSource, San Diego, CA, USA) according to the manufacturer’s instructions.

### Cell culture

Rat mesangial cells were obtained from American Type Culture Collection (ATCC, CRL-2573, Manassas, VA, USA) and cultured in Dulbecco's modified Eagle's medium containing 15% fetal bovine serum, 1% penicillin-streptomycin and G418 (0.4 mg/mL) according to ATCC recommendations.

### Measurement of AGEs formation

Rat mesangial cells were treated with 1 mM MGO, 10 nM Ex-4, or both for 10 h after synchronization with 1% fetal bovine serum for 13–16 h. AGEs formation was performed as previously described [51, 54]. Briefly, the cells were incubated in a mixture of chloroform and methanol (2:1 v/v) overnight followed by homogenization in 0.1 N NaOH and centrifugation at 8000*g* for 15 min at 4°C. AGEs formation in the supernatant was analyzed at an excitation/emission wavelength of 370/440 nm against 0.1 N NaOH as a blank and 1 mg/mL of bovine serum albumin in 0.1 N NaOH as a reference.

### Treatment of recombinant DPP4 peptide

Rat mesangial cells were seeded into 6-well plates at 5 × 10^4^ cells/well and treated with 1 mM MGO, 500 ng/mL DPP4 (#954-SE-010, R&D systems, MN, USA), or both for 10 h after synchronization with 1% fetal bovine serum for 13–16 h.

### D-Lactate assay

Rat mesangial cells were seeded into 6-well plates at 5 × 10^4^ cells/well and treated with 1 mM MGO, 10 nM Ex-4, or both for 10 h after synchronization with 1% fetal bovine serum for 13–16 h. The D-lactate level was measured using D-Lactate assay kit (#ab83429, Abcam, Cambridge, UK) according to the manufacturer’s instructions.

### Small interfering RNA

Rat mesangial cells were seeded into 6-well plates at 5 × 10^4^ cells/well and then transfected with either siRNA control (SN-1002, Bioneer, South Korea) or siRNA GLO-1 using Lipofectamine RNAi MAX (Thermo Fisher Scientific, Waltham, MA, USA) for 24 h according to the manufacturer’s instructions. The cells were treated with 0.75 mM MGO and 10 nM Ex-4 for 4 h and then harvested for further analysis.

### Western blotting

Total protein was isolated using mammalian protein extract buffer (28-9712-79, GE Life Sciences, Little Chalfont, UK) containing protease inhibitor cocktail (P8340, Sigma, St. Louis, MO, USA). The cytosol and nuclear fraction were prepared as described previously [55, 56]. An equal amount of protein was separated by sodium dodecyl sulfate polyacrylamide gel electrophoresis and transferred on polyvinylidene fluoride membranes. The membrane was blocked with blocking buffer for 1 h and then incubated serially with appropriate primary and secondary antibodies. Signals were detected by using an enhanced chemiluminescent detection system (Millipore, Billerica, MA, USA). The band density was quantified with the ImageJ program and normalized to actin, glyceraldehyde 3-phosphate dehydrogenase (GAPDH) and Lamin A/C. The antibodies used were as follows: anti-actin (#8457, Cell Signaling Technology, Danvers, MA, USA), anti-GAPDH (#MAB374, Millipore), anti-RAGE (sc-365154, Santa Cruz Biotechnology, Dallas, TX, USA), anti-DPP4/CD26 (5E8) (sc-8422, Santa Cruz), anti-glyoxalase-1 (sc-101537, Santa Cruz), anti-transforming growth factor (TGF)-β (#3711, Cell Signaling Technology), anti-Lamin A/C (#4777, Cell Signaling Technology, Danvers, MA, USA), anti-nuclear factor-erythroid 2 p45 subunit-related factor-2 (Nrf-2) (ab137550, Abcam), anti-monocyte chemoattractant protein 1 (MCP-1) (ab25124, Abcam), and anti-interleukin 6 (IL-6) (ab9324, Abcam).

### Gene expression analysis by RT-qPCR

Total RNA was isolated from rat kidney tissue or rat mesangial cells using RNAiso reagent (Takara, Shiga, Japan). The cDNA was synthesized with 2 μg of total RNA using the PrimeScript 1^st^ strand cDNA synthesis kit (6110A, Takara) according to the manufacturer’s instructions. Quantitative real-time PCR was performed using Applied Biosystem Prism 7900HT Real-Time PCR (Foster City, CA, USA) as previously reported [51]. The primers used are listed in Supplementary Table 1.

### Statistical analysis

Data are expressed as the means ± SEM. Statistical analysis was performed by one-way analysis of variance followed by Tukey’s post-hoc multiple comparison tests for more than two groups. An unpaired 2-tailed *t*-test was used to analyze two groups. Significance was considered when *p* values < 0.05.

## Supplementary Material

Supplementary Figures

Supplementary Table 1
