# Supplementary material for: Attenuation of diabetic kidney injury in DPP4-deficient rats; role of GLP-1 on the suppression of AGE formation by inducing glyoxalase 1
Source: Aging (Albany NY). 2020 Jan 6;12(1):593–610. doi: 10.18632/aging.102643 (PMC6977656; doi:10.18632/aging.102643)
Supplement: Supplementary Figures [file aging-12-102643-s002..pdf]

SUPPLEMENTARY FIGURES

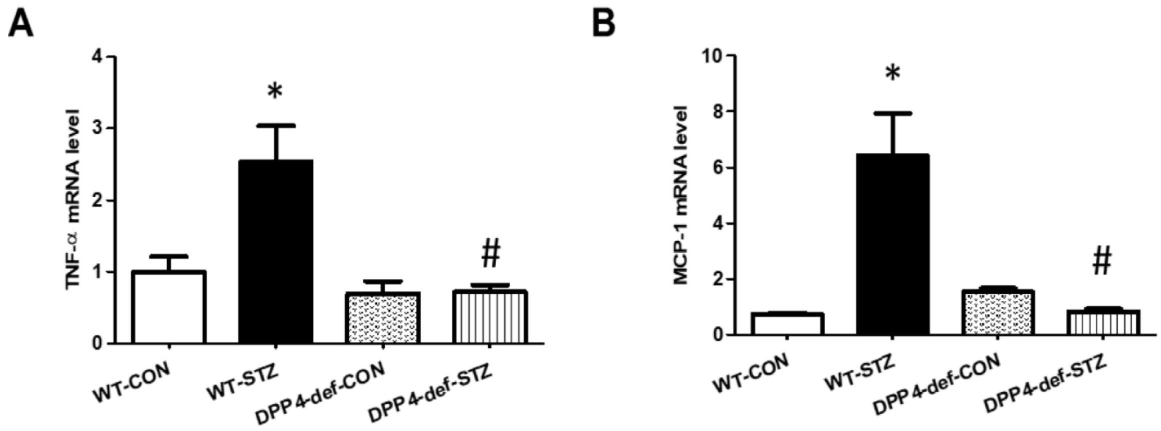

Supplementary Figure 1. The expression of inflammatory cytokines are reduced in STZ-induced diabetic rats. (**A**) *TNF-*α mRNA level, (**B**) *MCP-1* mRNA level in kidney tissues. Data are shown as the means ± SEM *$p < 0.05$ vs. WT-CON; $^{\#}p < 0.05$ vs. WT-STZ. n = 5–8 per group.

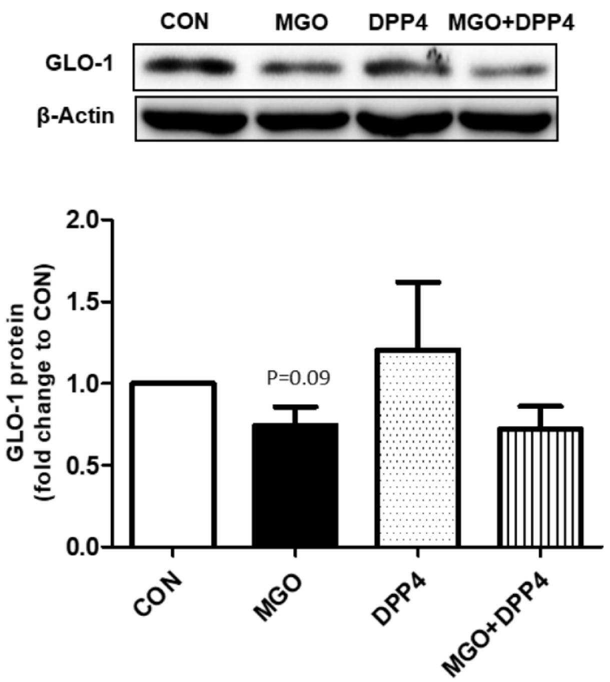

Supplementary Figure 2. Recombinant DPP4 protein treatment does not alter the expression of GLO-1 in rat mesangial cells. Rat mesangial cells were treated either with 1 mM MGO, 500 ng/mL recombinant DPP4, or both for 10 h after synchronization with 1% fetal bovine serum for 13-16 h. GLO-1 protein level with a representative blot. Data are shown as the means ± SEM. *P = 0.09* and CON, n = 3.

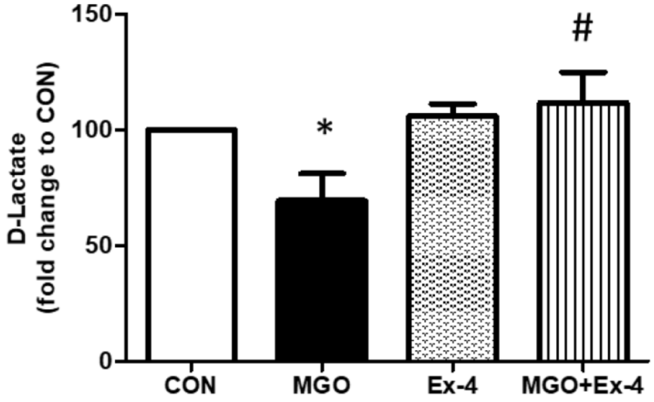

Supplementary Figure 3. D-lactate level in rat mesangial cells. Rat mesangial cells were treated either with 1 mM MGO, 10 nM Ex-4, or both for 10 h after synchronization with 1% fetal bovine serum for 13-16 h. The D-lactate level was measured using the D-lactate assay kit, according to the manufacturer's instructions. Data are shown as the means ± SEM. *$p < 0.05$ and CON, $^{\#}p < 0.05$ and MGO, n = 5.
