# Supplementary material for: Attenuation of diabetic kidney injury in DPP4-deficient rats; role of GLP-1 on the suppression of AGE formation by inducing glyoxalase 1
Source: Aging (Albany NY). 2020 Jan 6;12(1):593–610. doi: 10.18632/aging.102643 (PMC6977656; doi:10.18632/aging.102643)
Supplement: Supplementary Table 1 [file aging-12-102643-s001..pdf]

SUPPLEMENTARY TABLE

Supplementary Table 1. Primer Sequences.

Gene name	Direction	Sequence 5′-3′
RAGE	Forward	5′-GTG GGG ACA TGT GTG TCA GAG GGA A-3′
	Reverse	5′-TGA GGA GAG GGC TGG GCA GGG ACT-3′
GLO-1	Forward	5′-ATG CGA CCC AGA GTT ACC AC-3′
	Reverse	5′-CCA GGC CTT TCA TTT TAC CA-3′
TNF-α	Forward	5′-CAG CCG ATT TGC CAT TTC A-3′
	Reverse	5′-AGG GCT CTT GAT GGC AGA GA-3′
IL-6	Forward	5′-TCT CTC CGC AAG AGA CTT CCA-3′
	Reverse	5′-ATA CTG GTC TGT TGT GGG TGG-3′
MCP-1	Forward	5′-GTG CTG ACC CCA ATA AGG AA-3′
	Reverse	5′-TGA GGT GGT TGT GGA AAA GA-3′
TGF-β	Forward	5′-AGT CCT TTA GGG CGG TCA AT-3′
	Reverse	5′-TGG GAC TGA TCC CAT TGA TT-3′
Fibronectin	Forward	5′-GTG GCT GCC TTC AAC TTC TC-3′
	Reverse	5′-AGT CCT TTA GGG CGG TCA AT-3′
Cyclophilin	Forward	5′-TGC CAT CGC CAA GGA GTA G-3′
	Reverse	5′-TGC ACA GAC GGT CAC TCA AA-3′
GAPDH	Forward	5′-TGG TCT ACA TGT TCC AGT ATG ACT-3′
	Reverse	5′-CCA TTT GAT GTT AGC GGG ATC TC-3′
